# Transient left bundle branch block after posture change to the prone position during general anesthesia: A case report

**DOI:** 10.1097/MD.0000000000025190

**Published:** 2021-03-19

**Authors:** Hyun-Cheol Ko, Yong-Hyun Cho, Won Jang, Sun-Hee Kim, Hyun-Seok Lee, Woo-Hyeong Ko

**Affiliations:** Department of Anesthesiology and Pain Medicine, Seoul Sacred Heart General Hospital, Seoul, South Korea.

**Keywords:** hemodynamic changes, left bundle branch block, prone position

## Abstract

**Rationale:**

The prone position is commonly used in spinal surgery. There have been many studies on hemodynamic changes in the prone position during general anesthesia. We report a rare case of transient left bundle branch block (LBBB) in a prone position.

**Patient concern:**

Electrocardiogram (ECG) of a 64-year-old man scheduled for spinal surgery showed normal sinus rhythm change to LBBB after posture change to the prone position.

**Diagnosis:**

Twelve lead ECG revealed LBBB. His coronary angio-computed tomography results showed right coronary artery with 30% to 40% stenosis and left circumflex artery with 40% to 50% stenosis. The patient was diagnosed with stable angina and second-degree atrioventricular block of Mobitz type II.

**Intervention:**

Nitroglycerin was administered intravenously during surgery. Adequate oxygen was supplied to the patient. After surgery, the patient was prescribed clopidogrel, statins, angiotensin II receptor blocker, and a permanent pacemaker was inserted.

**Outcome:**

Surgery was completed without complications. After surgery, the transient LBBB changed to a normal sinus rhythm. The patient did not complain of chest pain or dyspnea.

**Lesson:**

The prone position causes significant hemodynamic changes. A high risk of cardiovascular disease may cause ischemic heart disease and ECG changes. Therefore, careful management is necessary.

## 1 Introduction

The prevalence of left bundle branch block (LBBB) is 0.06% to 0.1% of the general population, but it is associated with cardiovascular disease and increases patient mortality.^[1]^ It is caused by conduction block at both the anterior and posterior fascicles or at the upper main branch. Transient LBBB can be caused by various causes. Dilated cardiomyopathy, ischemic heart disease, changes in heart rate, acute pulmonary embolism, rheumatic heart disease, and increased intrathoracic pressure have been reported as causes.^[2–4]^ Although transient LBBB is a benign disease and has no significant effect on left ventricular performance, it can progress to permanent LBBB.^[2,5]^

LBBB during general anesthesia should be observed with caution because the patient cannot complain of symptoms and the prognosis is poor. During anesthesia, changes in ECG are relatively common, but changes in the LBBB are rare. In addition, there are no cases of change in LBBB during general anesthesia after changing from supine to prone position. In this case, the patient's ECG changed from normal sinus rhythm (NSR) to LBBB after the position changed to the prone position.

## 2 Case report

A 65-year-old Asian man (173 cm/90 kg) was admitted with lower back pain that occurred 2 years prior. Degenerative spondylolisthesis at L4-5 was found on magnetic resonance imaging (MRI) and posterior lumbar interbody fusion L4-5 was scheduled.

He had a history of hypertension, diabetes mellitus, and chronic obstructive pulmonary disease. His medications were calcium channel blockers and beta-blockers for hypertension

**Figure 1:**
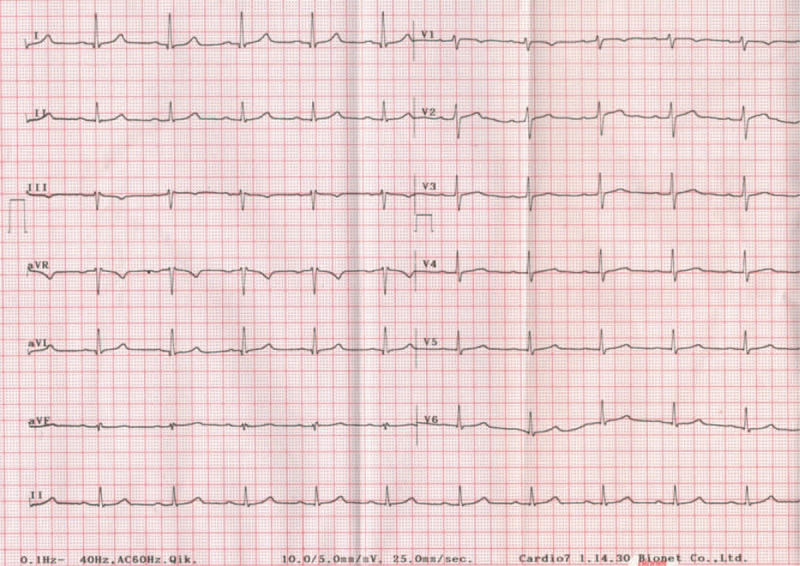
Preoperative ECG in ward.

On preoperative examination, there were no abnormalities in blood tests including cardiac markers performed before surgery, and ECG (Fig. 1) and chest imaging were normal. On echocardiography, the ejection fraction was 59%, and other findings were normal except for diastolic dysfunction grade 2.

Glycopylorate (0.2 mg) was administered intravenously for premedication before surgery. The patient's vital signs were monitored through invasive arterial blood pressure (BP) monitoring using radial artery, ECG, capnometry, and pulse oximetry equipment. In the operating room, his initial BP, heart rate (HR), Sp02 were 164/91 mmHg, 78 beats/min and 98%, respectively. ECG was NSR. Oxygen (100%) was delivered for preoxygenation prior to anesthetic induction. General anesthesia was induced with 150 mg of propofol and isoflurane (INH) in oxygen. Five minute after the administration of 70 mg of rocuromium, tracheal intubation was performed. General anesthesia was maintained with INH (1 MAC), O2 at 2 L/min, nitrous oxide at 2 L/min. The right radial artery was cannulated for invasive arterial BP monitoring and frequent sampling.

The patient's vital signs before posture change for surgery were BP 110/70 mmHg and HR 75 beats/min. Five minutes after changing position from supine to prone, his vital signs were BP 95/62 mmHg, HR 80 beats/min, and QRS widening appeared in lead II.

**Figure 2:**
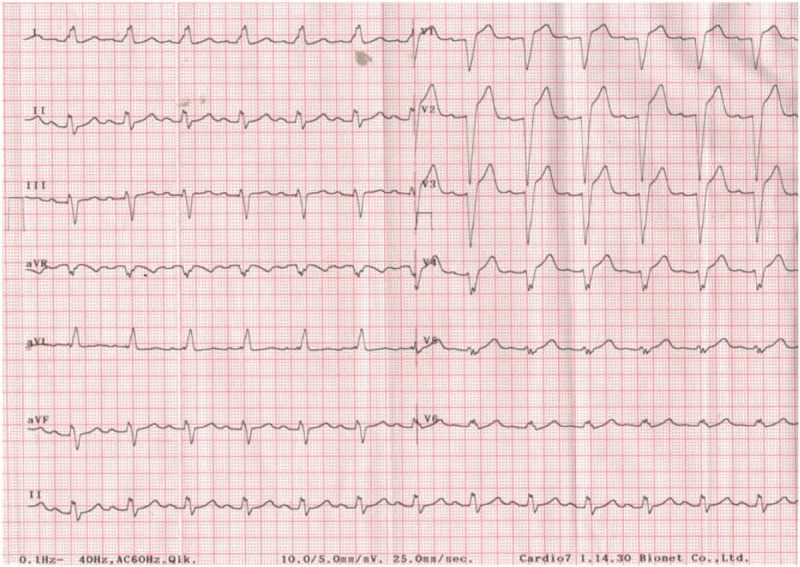
ECG immediately after changing from prone position to supine position. 12 lead ECG reveals LBBB.

To prepare for emergencies such as acute myocardial infarction and cardiac arrest, the patient was placed in the supine position, and a 12 lead ECG was taken immediately (Fig. 2). QRS widening (0.174 ms), absence of Q wave in lead I and V6, and monomorphic R wave V1-4 appeared in 12 lead ECG. Five minutes after changing the position to supine, his vital signs were BP 118/73 mmHg, HR 73 beats/min, and ECG changed to NSR.

NSR and stable vital signs were observed for 20 minutes, and continuous 1.0 μg/kg/min nitroglycerin (NTG) was injected intravenously under the advice of a cardiologist. After the posture changed to the prone position, LBBB appeared again in lead II, the operation was performed with the LBBB maintained and vital signs were stable. The operation was completed without complications. After the operation, the ECG was normal in the post-anesthesia care unit. The patient complained of back pain only, and there was no chest pain or dyspnea.

The patient was transferred to the intensive care unit, and a cardiac marker test was performed 3 times every 8 hours, and Holter tests were also performed for 24 hours. Cardiac markers were normal, and 24-hour Holter test results were transient right bundle branch block, LBBB, second degree atrioventricular (AV) block Mobitz type II. After two weeks of treatment, the patient was discharged and visited a tertiary hospital for a detailed cardiac examination. His coronary angio-computed tomography results showed right coronary artery with 30%-40% stenosis and left circumflex artery with 40%–50% stenosis. He was diagnosed with stable angina and a second-degree AV block Mobitz type II. He was prescribed clopidogrel, statins, angiotensin II receptor blockers, and a permanent pacemaker was inserted. The patient provided informed consent for the publication of the case, and this case followed the CARE guidelines.^[6]^

## 3 Discussion

The prone position is used for surgery on the back of the body, such as the lower extremities, back, and anus. The change from the supine position to the prone position causes many changes in the hemodynamic and respiratory systems, and studies on these changes have been reported. According to Yokoyama et al., the prone position and supportive frame for operation played an important role in the patient's hemodynamics.^[7]^ They found that the prone position using a convex saddle frame caused a significant reduction in cardiac index (CI, 17%), stroke volume index (SVI, 18%), and a significant increase in the systemic vascular resistance index (SVRI, 19%) compared with the supine position, but there were no significant hemodynamic changes in the flat prone position. This is due to peripheral blood pooling to the head and four extremities located lower than the heart, thereby reducing venous return. Dharmavaram et al. studied hemodynamic changes according to the type of supportive frames.^[8]^ They compared with Siemens, Andrews, Wilson frame, Jackson spine table, and longitudinal bolster using echocardiography. They found that all supportive frames caused a reduction in CI and SVI secondary to diminished preload and increased afterload, but the Jackson spine table produced the least effect on hemodynamic changes. Because of its use, the abdomen is totally unobstructed, and the head and four extremities are at the heart level, allowing adequate venous return. There are differences in the hemodynamic changes in the prone position depending on the anesthesia method. Sudheer et al. compared the CI when prone was induced in general anesthesia by total intravenous (TIVA) with propofol and general anesthesia by inhalation with INH.^[9]^ CI decreased significantly in the TIVA group (reduced by 25.9% compared to baseline) than in the INH group (reduced 12.9% compared to baseline).

We believe that the reasons for the transient LBBB in our case were increased thoracic pressure and decreased CI. In the case reported by Chow et al., coughing and laughter increased intrathoracic pressure-induced transient LBBB.^[4]^ They suggested that increased intrathoracic pressure due to cough and laughter reduces cardiac perfusion. Reduced cardiac perfusion and arteriosclerotic heart disease lead to ischemic heart disease. In fact, Johnson et al found that arteriosclerotic heart disease and hypertension were the main clinical features of LBBB.^[10]^

In our case, after general anesthesia, many hemodynamic changes occurred after changing to the prone position during spine surgery using the Wilson frame. Venous return was reduced due to peripheral blood pooling; therefore, the preload was reduced. Increased intrathoracic pressure and decreased left ventricle compliance increased afterload, which can be considered to decrease heart diastolic function and arterial filling.^[11]^ In addition, baroreceptors are inhibited by CI reduction, and sympathetic activity is increased.^[12]^ In our patient with coronary artery stenosis, the patient was susceptible to ischemic hemodynamic changes, and reversible myocardial ischemia occurred due to decreased diastolic function and increased sympathetic activity due to various outcomes, which is thought to result in transient LBBB. The rate-dependent LBBB was excluded because the LBBB was maintained during surgery and the HR was similar to that in the supine position. Acute pulmonary embolism-induced LBBB was also excluded because the heart beat was normal during surgery, and the D-dimer test results were normal.

## 4 Conclusion

In conclusion, if a patient with a high risk of cardiovascular disease undergoes surgery in the prone posture after general anesthesia, dramatic hemodynamic changes may occur. In addition, because the patient cannot complain of symptoms, ECG and hemodynamic changes must be carefully monitored. In patients with high cardiovascular risk, inhalation anesthesia, sufficient fluid resuscitation, using a longitudinal bolster and a Jackson knife table may minimize hemodynamic changes and reduce ischemic damage to the heart.^[8]^ In addition, when a new onset of LBBB occurs after prone posture, the patient should be treated as having ischemic heart disease until the cause is found. NTG should be administered with the advice of a cardiologist. The fraction of inspired oxygen should be increased to ensure adequate myocardial oxygen demand, and hemoglobin level should be kept above 10 mg/dl. Further evaluations of the heart, such as echocardiography, coronary angiography, and myocardial perfusion scans are needed. Although this case report showed that prone position can cause myocardial ischemic damage and ECG change in high risk of cardiovascular patients, it is limited that there are not many studies on myocardial ischemic change according to quantitative change of intrathoracic pressure.

## Author contributions

**Conceptualization:** Hyun Cheol Ko, Yong-Hyun Cho, Hyun-Seok Lee.

**Supervision:** Won Jang.

**Visualization:** Sun-Hee Kim, Woo-Hyeong Ko.

**Writing – original draft:** Hyun Cheol Ko.

**Writing – review & editing:** Hyun Cheol Ko, Yong-Hyun Cho.
